# MRAC Control with Prior Model Knowledge for Asymmetric Damaged Aircraft

**DOI:** 10.1155/2015/247301

**Published:** 2015-06-09

**Authors:** Xieyu Xu, Lingyu Yang, Jing Zhang

**Affiliations:** ^1^CNIGC Institution of Navigation and Control, Chedaogou No. 10, Haidian District, Beijing 100191, China; ^2^Department of Automation Science and Electrical Engineering, Beihang University, Haidian District, Beijing 100191, China

## Abstract

This paper develops a novel state-tracking multivariable model reference adaptive control (MRAC) technique utilizing prior knowledge of plant models to recover control performance of an asymmetric structural damaged aircraft. A modification of linear model representation is given. With prior knowledge on structural damage, a polytope linear parameter varying (LPV) model is derived to cover all concerned damage conditions. An MRAC method is developed for the polytope model, of which the stability and asymptotic error convergence are theoretically proved. The proposed technique reduces the number of parameters to be adapted and thus decreases computational cost and requires less input information. The method is validated by simulations on NASA generic transport model (GTM) with damage.

## 1. Introduction

It has been shown that the vehicle damage to airframe and engines had led to quite a few aircraft accidents and fatalities in recent years [1]. Asymmetric aircraft structural damage results in abrupt deterioration on flight performance and handling quality, which impairs safety. Researches to recover aircraft stability and handling qualities, like fault-tolerant flight control, reconfigurable control method, and so forth, have already become a hot spot in related academic fields.

Lots of studies have been conducted to recover control performance under aircraft failures or damages. NASA has launched several aircraft safety projects like IFCS [2] and AvSP [3], which innovate flight control algorithms and experiment on aircraft testbeds. Various flight control laws like the adaptive control designs [4–9], linear quadratic regulator (LQR) designs [6, 10, 11], and robust linear designs [12] were proposed and evaluated by flights. The pilot Cooper-Harper ratings showed that the proposed methods could retain the handling qualities under certain failure cases [4, 13].

Generally, the adaptive control algorithms are quite popular in this field. Reference [14] used an adaptive artificial neural network (ANN) control method to recover the handling qualities of an F-18 model. The method uses a pretrained ANN to provide a model inversion block with aerodynamics and handling characteristics and another online-adjusted ANN to compensate modeling error and failure conditions. Reference [15] extended the result of [14]. It changes the pretrained ANN to an online learning network, which adjusts the inversion block adaptively, resulting in a hybrid direct and indirect adaptive ANN control scheme. Reference [16] designed an angular rate controller using adaptive single hidden layer ANN method for an unmanned airborne vehicle. With a classic linear guidance law, several successful automated landings were made in flight experiments with 25% left wing loss.

Except for ANN adaptive laws, multivariable MRAC with various structures were also studied in this field. Reference [9] combined a direct MRAC method with a parameter estimator using gradient algorithm, obtaining a combined MRAC structure. References [6, 17] proposed a derivative-free MRAC using delayed weight update law. Reference [7] utilized the *L*_1_ adaptive law [18] for a flight controller design. Reference [19] analyzed the plant characteristics by LDS decomposition of the high frequency gain matrix (HFGM) of the damaged aircraft. Realizing the signs of the leading principle minors of the HFGM does not change before and after damage. Along with several other results and assumptions, an MRAC law with output feedback structure is proposed. The method is validated through simulations on a high-fidelity GTM model with left wing tip damage. A similar design can be found in [20], which uses a state feedback structure with adaptive gains instead of the original output feedback structure.

The methods mentioned above do not need to take the characteristics of damage conditions into account. As long as the assumptions hold, these methods can be applied to various damage cases and allow for relatively large parameter changes. However, the range of admissible parameter changes can be so large, in a way that is far enough to cover all damage cases. If proper descriptions can be used to model the concerned damage cases, it is possible to design a controller with parameters adjusted in a smaller region, reducing computation workload and improving performance.

Based on the analysis above, this paper models the structural damaged aircraft with polytope LPV models and model reference controllers (MRCs) are given, which explicitly integrates plant characteristics. The parameters updated by adaptive laws are the polytope interpolation coefficients, instead of control gains in traditional MRAC designs. The number of the adaptive parameters is therefore reduced. The paper is organized as follows.  Section 1 introduces the background and design philosophy of the proposed method.  Section 2 models and analyzes a damaged aircraft, namely, GTM. A polytope linear model is introduced to describe the concerned damage cases.  Section 3 designs the control laws and adaptive laws, as well as Lyapunov stability and error convergence proof.  Section 4 validates the design by simulations on a 6DOF nonlinear GTM model with left outboard wing tip damage.  Section 5 concludes the whole paper.

## 2. Modeling of an Asymmetric Damaged Aircraft with Prior Knowledge

### 2.1. Nonlinear 6DOF Modeling

This paper studies the GTM from IRAC project in AvSP program carried out by NASA. Due to the limitation of modeling data, this paper only discusses the damage case of left outboard wing tip loss. With data and methods from [21], the characteristics of aerodynamics, mass, inertia, center of gravity (CG) movement, and so forth, can be modeled. With methods from [22], the rigid body dynamic equations are deduced. It should be noted that the sensors remain unmoved before and after damage, so that the point which the dynamic equations describe remains unchanged. The dynamic equation can be written as follows:

(1) FM=mI−mΔssmΔssIV˙ω˙ +mωss−mωssΔssmωssΔssωssI−mVssΔssVω.

*F* and *M* refer to the total force and moment relevant to original CG, respectively. *m*, *I*, and Δ are the total mass, inertia in original CG, and the offset of CG reference to its origin location. *V* = [*u*, *v*, *w*]^*T*^, *ω* = [*p*, *q*, *r*]^*T*^ are the line speed and angular speed described in the origin body frame of the aircraft. Superscript ^*ss*^ is the skew-symmetric matrix form of a vector, (*x* ^*ss*^)*y* = *x* × *y*.

The line speed of a specific point generally changes with its location, only except that it moves parallel to the rotation axis of the rigid body. The calculation of the line acceleration has to take the centripetal and the Coriolis force into account, as long as the point is not located on the rotation axis. In short, due to the CG movement by the structural damage, the calculation of line speed becomes quite complicated, coupling with angular speed and angular acceleration. However, if the body frame is selected parallel to the original one, the direction of each axis does not change, and the values of the angular speed are the same wherever the frame is. Thus, the angular speed can be modeled in the body frame at the new CG without changing its value, while the line speed has to be modeled at the same point. Rewrite (1) as follows:

(2) mI−mΔss0I−V˙ω˙ =−mωssmωssΔss0−ωssI−Vω+FM−.

Since the value of angular speed remains unchanged, the symbol *ω* is used as before. $\overset{-}{I}$ and $\overset{-}{M}$ are the inertia and moment represented in the new body frame located at CG after damage. The kinetic equations are the same as those of normal aircrafts. [*ϕ*, *θ*, *ψ*] is the Euler angle. [*x*_*g*_, *y*_*g*_, *z*_*g*_] is the position vector relative to the ground:

(3) $$\begin{array}{c} \left[\begin{array}{c} \dot{\phi }\\ \dot{\theta }\\ \dot{\psi } \end{array}\right]=\left[\begin{array}{ccc} 1 & tan\theta sin\phi & tan\theta cos\phi \\ 0 & cos\phi & -sin\phi \\ 0 & \sin\phi \sec\theta & \cos\phi \sec\theta \end{array}\right]\left[\begin{array}{c} p\\ q\\ r \end{array}\right],\\ \left[\begin{array}{c} {\dot{x}}_{g}\\ {\dot{y}}_{g}\\ {\dot{z}}_{g} \end{array}\right]=\left[\begin{array}{ccc} cos\theta cos\psi & sin\phi sin\theta cos\psi -cos\phi sin\psi & sin\phi sin\psi +cos\phi sin\theta cos\psi \\ cos\theta sin\psi & sin\phi sin\theta sin\psi +cos\phi cos\psi & -sin\phi cos\psi +cos\phi sin\theta sin\psi \\ -sin\theta & sin\phi cos\theta & cos\phi cos\theta \end{array}\right]\left[\begin{array}{c} u\\ v\\ w \end{array}\right]. \end{array}$$

With the sensors unmoved, the definitions of airspeed *V*, angle of attack *α*, and sideslip *β* are the same as before. The equations are as follows:

(4) $$\begin{array}{c} \alpha =arctan\frac{w}{u},\\ \beta =arcsin\frac{v}{V},\\ V={\left\|V\right\|}_{2}. \end{array}$$

### 2.2. Polytope Linear Model

Generally, the nonlinear equations of an aircraft can be described as

(5) $$\begin{array}{c} \dot{x}\left(t\right)=f\left(x\left(t\right),u\left(t\right)\right). \end{array}$$

*x*, *u* represent the state and input vectors, respectively. An operation point, or trim point, should be selected before linearization. It should be noted that the actual damage cases are unknown, which renders the trim point unavailable. A general solution is to select a common operation point for all damage cases, for example, the normal point (*x*_0_, *u*_0_) [20]. Because the normal point generally is not the trim point for damaged cases, an extra term *f*_*i*_ is added to the state-space equations, as follows [23]:

(6) Δx˙t=∑AiχitΔxt+∑BiχitΔut +∑fiχitχit=1if  At,Bt,ft=Ai,Bi,fi0otherwise,

where *A*_*i*_ ∈ *ℝ* ^*n*×*n*^, *B*_*i*_ ∈ *ℝ* ^*n*×*m*^ are unknown constant matrices, representing the state transition matrices and control matrices of different patterns and degrees of structural damage, respectively. *f*_*i*_ ∈ *ℝ* ^*n*^ is the unknown constant disturbance. Various control methods can be designed with (6) [20, 23]. However, *A*_*i*_, *B*_*i*_, *f*_*i*_ are unknown constants with weak constraints like the signs of leading principle minors of high frequency gain matrix that do not change, which could lead to relatively large parameter variations. The actual damage cases may not cover all these variations. This paper believes that if proper definitions of different damage cases are introduced, the parameter variations can be limited, which facilitates the controller designs.

Based on the above analysis, the model of damaged aircraft is rewritten in a polytope form:

(7) $$\begin{array}{c} \Delta \dot{x}=A\left(p\right)\Delta x+B\left(p\right)\Delta u+f\left(p\right),\\ \left[A\left(p\right),B\left(p\right)\right]\in \Omega =Co\left\{\left[A_{1}^{*},B_{1}^{*}\right],\ldots ,\left[A_{l}^{*},B_{l}^{*}\right]\right\}, \end{array}$$

where [*A*_*i*_^*∗*^, *B*_*i*_^*∗*^], *i* = 1,…, *l* are known constant matrices, the vertices of polytope *Ω*. There exist nonnegative coefficients *α*_*i*_(*p*); thus, [*A*(*p*), *B*(*p*)] can be calculated by

(8) $$\begin{array}{c} \left[A\left(p\right),B\left(p\right)\right]=\overset{l}{\underset{i=1}{\sum }}\alpha_{i}\left(p\right)\left[A_{i}^{*},B_{i}^{*}\right],\\ \overset{l}{\underset{i=1}{\sum }}\alpha_{i}\left(p\right)=1,\;0\leq \alpha_{i}\left(p\right)\leq 1. \end{array}$$

Parameter *p* represents different damage patterns and degrees. For any specific damage case, it is assumed that a certain *p* ^*∗*^ can describe the model of the damaged aircraft. *f*(*p*) is the extra unknown constant term, for the aircraft is untrimmed under damage. *f*(*p*) also captures other effects from structural damages and is not modelled in the polytope form. Finally the model is written as

(9) $$\begin{array}{c} \Delta \dot{x}=\overset{l}{\underset{i=1}{\sum }}\alpha_{i}\left(p\right)A_{i}^{*}\Delta x+\overset{l}{\underset{i=1}{\sum }}\alpha_{i}\left(p\right)B_{i}^{*}\Delta u+f\left(p\right). \end{array}$$

Compared to (6), the only unknown variables in (9) are *α* = [*α*_1_,…,*α*_*l*_]^*T*^ ∈ *ℝ* ^*l*^ and *f*(*p*) ∈ *ℝ* ^*n*^. Different damage cases simply differs in weighting coefficient *α* and constant *f*(*p*), thus vastly reduces the number of unknown variables.

### 2.3. Vertex Calculation by High Order SVD Method

As stated before, the less the number of vertices is, the less complicated the controller is. It is crucial to balance between the accuracy and the complexity of the polytope aircraft model with damage of various patterns and degrees. The high order SVD (HOSVD) method is a decomposition method for high order arrays or data tensors. By representing the polytope LTI models into a tensor-product (TP) form, the HOSVD method can be used to reduce the number of vertices [24].

First, a parameter grid is generated on the concerned damage cases. For the left outboard wing tip damage case, a grid of *k*-element vector is used. Each node of the grid represents a different wing tip loss ratio to the semispan. The GTM model with damage is linearized at each node of the grid, written in the system matrix form

(10) $$\begin{array}{c} S_{i}=\left[\begin{array}{cc} A_{i} & B_{i}\\ C_{i} & D_{i} \end{array}\right]. \end{array}$$

For every specific case in the concerned damage cases, the model *S*(*p*) can be written as the interpolation of the linearized models. That is,

(11) $$\begin{array}{c} S\left(p\right)=\sum \alpha_{i}\left(p\right)S_{i}, \end{array}$$

wherein function *α*_*i*_(*p*) is the interpolation coefficient function. The interpolation calculation can be rewritten in a compact TP form. Stack all the linearized models to make the model tensor *𝒮* and all the coefficients to make the parameter tensor *𝒰*. Thus,

(12) $$\begin{array}{c} S\left(p\right)=\sum \alpha_{i}\left(p\right)S_{i}=S⊗U\left(p\right). \end{array}$$

To accurately describe the original model, a relatively intense grid has to be generated. The algorithm based on HOSVD method from [24] is adopted in this paper to simplify the grid. By omission of small singular values, the number of models and corresponding coefficients can be reduced, without losing accuracy to the original ones. With this algorithm, the preliminary TP model can be approximated to

(13) $$\begin{array}{c} S\left(p\right)=S⊗U\left(p\right)\approx S^{*}⊗U^{*}\left(p\right). \end{array}$$

New models *S*_*i*_^*∗*^ and interpolation coefficients *α*_*i*_^*∗*^(*p*), *i* = 1,…, *l*, can be extracted from the system tensor *𝒮* ^*∗*^ and the coefficient tensor *𝒰* ^*∗*^. The algorithm automatically processes the coefficients to build up a polytope model, constrained by ∑*α*_*i*_^*∗*^ = 1, *α*_*i*_^*∗*^ > 0.

The numerical results are shown as follows. The left outboard wing tip loss of GTM ranges from 0% to 33% semispan. A grid size of 10 is selected. State-space models are obtained by linearizing nonlinear GTM models of 1 normal case and 9 damage cases equally spaced along the loss ratios to wing span. After rewriting the models into TP form, the singular values calculated by HOSVD method are given as follows in sorted sequence:

(14) $$\begin{array}{c} \sigma =\left[\begin{array}{ccccc} 166.4366 & 0.1842 & 0.0035 & 0.0034 & \cdots \end{array}\right]. \end{array}$$

It can be seen that the first 2 singular values are relatively large. By omitting the rest of singular values, the algorithm simplifies the 10 models to 3 vertex models. Thus, any model from the original 10 models, *S*_*i*_, for example, can be approximated by interpolation of the obtained 3 models as follows:

(15) $$\begin{array}{c} S_{i}\approx {\hat{S}}_{i}=\overset{3}{\underset{j=1}{\sum }}\alpha_{j}^{*}\left(i\right)S_{j}^{*}. \end{array}$$

**Figure 1:**
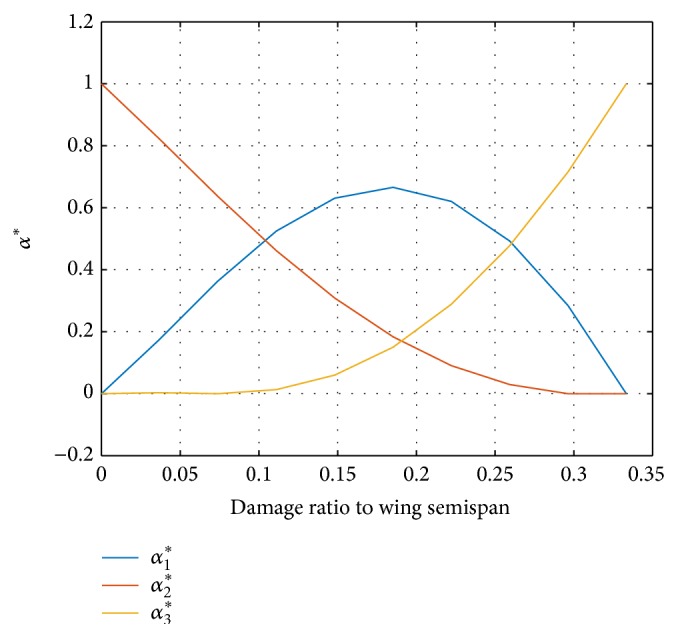
The curves of the interpolation coefficients to wing tip loss ratios.

*α*_*j*_^*∗*^(*i*) is the interpolation coefficient. The output models are in accordance with the properties of polytope models. The curves of the interpolation coefficients to wing tip loss ratios are shown in Figure 1.

**Figure 2:**
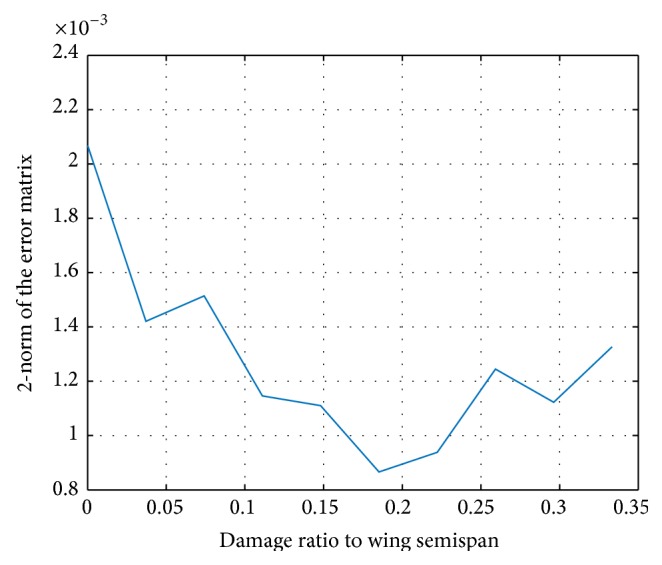
The accuracy of processed model.

The accuracy of the processing procedure can be measured by the 2-norm of the matrix error ${\tilde{S}}_{i}=S_{i}-{\hat{S}}_{i}$, as shown in Figure 2.

It can be shown that the error norm is relatively small, which concludes that the resulting polytope model is close to the original ones. The subsequent designs are based on the obtained polytope model.

The control algorithm proposed in this paper is not of LMI designs in [24]. The interpolation parameters satisfy ∑_*j*_ *α*_*j*_(*i*) = 1 only, which means extrapolation is admissible in the algorithm. The parameters are treated independent of each other, without restrictions in Figure 1. That is, the 3 parameters can vary freely on the plane ∑_*j*_ *α*_*j*_(*i*) = 1 in the 3D space, while Figure 1 restricts the parameters to a curve. As a result, it is expected that the polytope model can describe more damage cases by relaxing the restrictions on the parameters.

### 2.4. GTM Model Simplification

Assume the polytope model of GTM is written as

(16) $$\begin{array}{c} {\dot{x}}_{GTM}=A\left(p\right)x_{GTM}+B\left(p\right)u+f_{GTM}\left(p\right), \end{array}$$

wherein

(17) $$\begin{array}{c} x_{GTM}\left(t\right)={\left[q,p,r,V,\alpha ,\theta ,\beta ,\phi \right]}^{T},\\ u\left(t\right)={\left[\delta_{e},\delta_{a},\delta_{r}\right]}^{T}. \end{array}$$

The angular rates are to be controlled. Rewrite the state-space equations to treat angular rates as states, while the other signals related to angular rates are treated as measurable disturbances. Dividing the original states into two vectors yields

(18) $$\begin{array}{c} x_{GTM}^{T}=\left[x^{T},z^{T}\right],\\ x≜{\left[q,p,r\right]}^{T},\\ z≜{\left[V,\alpha ,\theta ,\beta ,\phi \right]}^{T}. \end{array}$$

Reformulate the state-space equation as follows:

(19) x˙z˙=AxxpAxzpAzxpAzzpxz +BxpBzpu+fxpfzp.

Finally, the polytope model with angular rates as states is shown below:

(20) $$\begin{array}{c} \dot{x}=A_{xx}\left(p\right)x+A_{xz}\left(p\right)z+B_{x}\left(p\right)u+f_{x}\left(p\right). \end{array}$$

The model is explained as follows. The states are defined as the angular rates. The state transition matrix is *A*_*xx*_. The input of the model is separated into 2 parts, the first part of which is the remaining state *z* with input matrix *A*_*xz*_. The second part is the original input *u* with input matrix *B*_*x*_, which describes the effects of control surfaces. Because the damaged aircraft is not trimmed, a constant disturbance *f*_*x*_(*p*) is added to the equation. For the convenience of the following discussions, (20) is rewritten as

(21) $$\begin{array}{c} \dot{x}=A\left(p\right)x+H\left(p\right)z+B\left(p\right)u+f\left(p\right). \end{array}$$

For the specific case of GTM, the linear model of the undamaged case is shown below:

(22) x˙=−3.0362000−4.65760.54810−0.3932−1.0176︸Ax +0−29.0039000000−75.2928000024.89980︸Hz +−43.266200039.568611.140903.2818−28.537︸Bu +000︸f0.

The linear model of 33% semispan left outboard wing tip loss is written as follows:

(23) x˙=−3.004−0.13460.0062−0.5033−3.21880.4941−0.0508−0.2771−1.0288︸Ax +2.2889−20.8150−0.71960−34.4495−158.34920−75.29280−3.7582−21.7562025.86890︸Hz +−43.31242.6631−0.04420.645522.046611.31550.02111.8451−28.7231︸Bu +53.0318−797.3925−86.6825︸f0.

It can be seen from the above results that the attitude angles, namely, *θ* and *ϕ*, have no effect on the angular rates, which can be omitted in the model. The omission is reasonable because the moment of aircraft can be generated only by aerodynamic moment and thrust, and the attitude angles have nothing to do with it. Thus, the state *z* is simplified to *z* = [*V*, *α*, *β*]^*T*^.

Although the input matrix *B* changes with damage, the characteristics of *B*, like the domination and signs of the diagonal elements, remain unchanged. The numeric changes of nondiagonal elements relative to diagonal ones can also be ignored. Based on the above conclusions, the *B* matrix is treated as a constant before and after wing tip damage. In short,

(24) $$\begin{array}{c} B\left(p\right)\approx B. \end{array}$$

For the convenience of design, the *B* matrix adopted in polytope model is the undamaged one.

## 3. The MRAC Design

Model reference scheme with state-tracking method is adopted to recover the control performance after the aircraft structural damage. The reference model is designed as follows:

(25) $$\begin{array}{c} {\dot{x}}_{m}=A_{m}x_{m}+B_{m}r. \end{array}$$

Hurwitz state transition matrix *A*_*m*_ and input matrix *B*_*m*_ reflect the desired dynamic performance. It is known that given symmetric positive real matrix *Q*, there exists a symmetric positive real matrix *P*, satisfying

(26) $$\begin{array}{c} A_{m}^{T}P+PA_{m}+Q=0. \end{array}$$

Considering the angular motion of an aircraft can be directly altered by control surfaces, input matrix *B* will have full rank to be invertible. The model can be rewritten as

(27) x˙=Amx+B ×u+B−1∑αiAi∗−Amx+B−1Hpz+B−1f.

Define

(28) $$\begin{array}{c} \mu_{i}≜\left[\begin{array}{cc} B^{-1}\left(A_{i}^{*}-A_{m}\right) & B^{-1}H_{i}^{*} \end{array}\right]\left[\begin{array}{c} x\\ z \end{array}\right],\\ \overset{-}{f}≜B^{-1}f. \end{array}$$

Thus,

(29) $$\begin{array}{c} \dot{x}=A_{m}x+B\left(u+\left[\begin{array}{ccc} \mu_{1} & \cdots & \mu_{l} \end{array}\right]\left[\begin{array}{c} \alpha_{1}\\ \vdots \\ \alpha_{l} \end{array}\right]+\overset{-}{f}\right). \end{array}$$

Rewrite the polytope constraint ∑_*i*=1_^*l*^ *α*_*i*_ = 1 to *α*_1_ = 1 − ∑_*i*=2_^*l*^ *α*_*i*_ and substitute

(30) x˙=Amx+B ×u+μ1+μ2−μ1⋯μl−μ1α2⋮αl+f−=Amx+Bu+μ1+M−α−+f−0.

It is assumed that every state of the aircraft can be measured. Matrix $\overset{-}{M}$ is known, since signal *μ*_*i*_ can be measured and calculated with known signals. The parameter vector $\overset{-}{\alpha }$ concatenated from *α*_2_ to *α*_*l*_ and the constant offset $\overset{-}{f}$ are unknown in actual damage cases. The control law can be designed as follows to alter the plant dynamic characteristics to reference model precisely, if every piece of parameter is known:

(31) $$\begin{array}{c} u=B^{-1}B_{m}r-\mu_{1}-\overset{-}{M}\overset{-}{\alpha }-\overset{-}{f}. \end{array}$$

However, $\overset{-}{\alpha }$ and $\overset{-}{f}$ are unknown after damage. With certain-equivalence design principle [25], the control law is designed as

(32) $$\begin{array}{c} u=B^{-1}B_{m}r-\mu_{1}-\overset{-}{M}\hat{\overset{-}{\alpha }}-\hat{\overset{-}{f}}. \end{array}$$

$\hat{\overset{-}{\alpha }}$ and $\hat{\overset{-}{f}}$ are estimates of $\overset{-}{\alpha }$ and $\overset{-}{f}$ by adaptive laws, respectively. Define the parameter errors as $\tilde{\overset{-}{\alpha }}≜\hat{\overset{-}{\alpha }}-\overset{-}{\alpha }$, $\tilde{\overset{-}{f}}≜\hat{\overset{-}{f}}-\overset{-}{f}$ and substitute the control law into (30), resulting in

(33) $$\begin{array}{c} \dot{x}=A_{m}x+B_{m}r-B\overset{-}{M}\tilde{\overset{-}{\alpha }}-B\tilde{\overset{-}{f}}. \end{array}$$

The tracking error is defined as *e*≜*x* − *x*_*m*_, the dynamics of which can be obtained as

(34) $$\begin{array}{c} \dot{e}=A_{m}e-B\overset{-}{M}\tilde{\overset{-}{\alpha }}-B\tilde{\overset{-}{f}}. \end{array}$$

The adaptive laws are designed using Lyapunov direct method. The Lyapunov function *V* is chosen to be

(35) $$\begin{array}{c} V=e^{T}Pe+{\tilde{\overset{-}{\alpha }}}^{T}{\Gamma_{\alpha }}^{-1}\tilde{\overset{-}{\alpha }}+{\tilde{\overset{-}{f}}}^{T}{\Gamma_{f}}^{-1}\tilde{\overset{-}{f}}. \end{array}$$

Γ_*α*_, Γ_*f*_ are positive symmetric matrices. The derivative of Lyapunov function *V* along the dynamics of system is

(36) V˙=e˙TPe+eTPe˙+2α−~˙TΓα−1α−~+2f−~˙TΓf−1f−~=−eTQe−2eTPBM−α−~−2eTPBf−~ +2α−~˙TΓα−1α−~+2f−~˙TΓf−1f−~.

The adaptive laws are selected as

(37) $$\begin{array}{c} {\dot{\tilde{\overset{-}{\alpha }}}}^{T}=e^{T}PB\overset{-}{M}\Gamma_{\alpha },\\ {\dot{\tilde{\overset{-}{f}}}}^{T}=e^{T}PB\Gamma_{f}. \end{array}$$

Thus, the derivate $\dot{V}$ becomes

(38) $$\begin{array}{c} \dot{V}=-e^{T}Qe. \end{array}$$

The design process of the adaptive law in this paper is quite similar to the classical ones using the Lyapunov method. Properties like stability and asymptotic error convergence can also be proved using same procedures. All states in the system are bounded due to the positivity of *V* and seminegativity of $\dot{V}$, so that the system is stable in the Lyapunov sense. The boundedness of states guarantees that $\ddot{V}$ is bounded, which means $\dot{V}$ is uniformly continuous. Using Barbalat's lemma, it can be concluded that $\dot{V}$ converges to zero asymptotically; that is, the tracking error *e* → 0. In summary, the properties of the proposed method are stated as follows.

**Theorem 1 .**

The MRAC theme with control law (32) updated by adaptive law (37), when applied to the plant (30), guarantees the closed-loop signal boundedness and asymptotic state-tracking *e* = *x* − *x*_*m*_ → 0.

**Remark 2 .**

Thanks to the popularity of the standard Lyapunov design procedure, lots of modifications can be applied to the control law, such as robust modifications [26], predictor-based or reference model modifications to improve transient performance [27–30], and adaptive law modifications [31].

**Remark 3 .**

The proposed method updates the interpolation coefficients of the feedback gains. The gains are designed beforehand for the vertices of the polytope model, which integrates prior information of different cases of damage into the control law. With prior knowledge of various damage cases, the performance of the design is expected to be improved. The HOSVD and model simplification process reduce the number of vertices and the amount of parameters. Less stimulation is required for the input signal [25].

**Remark 4 .**

The linearization of the original 6DOF nonlinear model and the vertex simplification using HOSVD method result in an approximated damaged aircraft model. There always exists modeling error between the real model of various damage cases and the developed polytope model. Similar to common adaptive algorithms, the update law has to be modified to ensure closed-loop stability [25]. Modifications like the projection operator have to be applied to the adaptive law in real cases.

**Remark 5 .**

It should be noted that the maximum deflection of control surfaces is finite in real aircrafts, which poses a constraint on *u* in (32) and is not taken into consideration in MRAC design process. It can be seen in (23) that some values in matrix *H* and vector *f*_0_ are quite large, which might saturate *u* and cause the aircraft to be uncontrollable in some cases. Actually, if all control surfaces are limited within ±30°, which is even larger than that in [21], it is impossible to maintain a steady cruise in the operation point of (23) in 33% left wing tip loss case. The steady cruise means that no rotation is allowed; thus, the angular speed [*p*, *q*, *r*] keeps zero, and the operation point means the other states in the linearization model (23), namely, [*V*, *α*, *β*], are equal to zero, resulting in

(39) −43.31242.6631−0.04420.645522.046611.31550.02111.8451−28.7231︸Bu +53.0318−797.3925−86.6825︸f0=0,u=−3.4640−36.41410.6762T.

It can be seen that the second element in *u* exceeds −30°, which means the aileron is saturated, concluding that it is impossible to balance the constant offset *f*_0_ solely by input *u*.

However, it is feasible to maintain a straight cruise under some attack angle *α* and sideslip *β*, and it is possible to saturate the control surfaces under improper *α* and *β*. Due to the left wing tip loss, if the damaged aircraft is flown with relative large lift force, the left roll aerodynamic moment will be too large to be compensated by ailerons. Detailed discussions of flight performance under various damage cases may be found in [32, 33] and are beyond the scope of this paper.

## 4. Simulation Study

In this section, the developed adaptive law under the obtained linear polytope model is validated by simulations on a NASA GTM nonlinear model with damage. GTM is a 5.5% dynamically scaled twin-turbine powered aircraft model, designed and manufactured in the NASA AvSP program, dedicated to flight testing of research control laws in adverse flight conditions [34].

**Figure 3:**
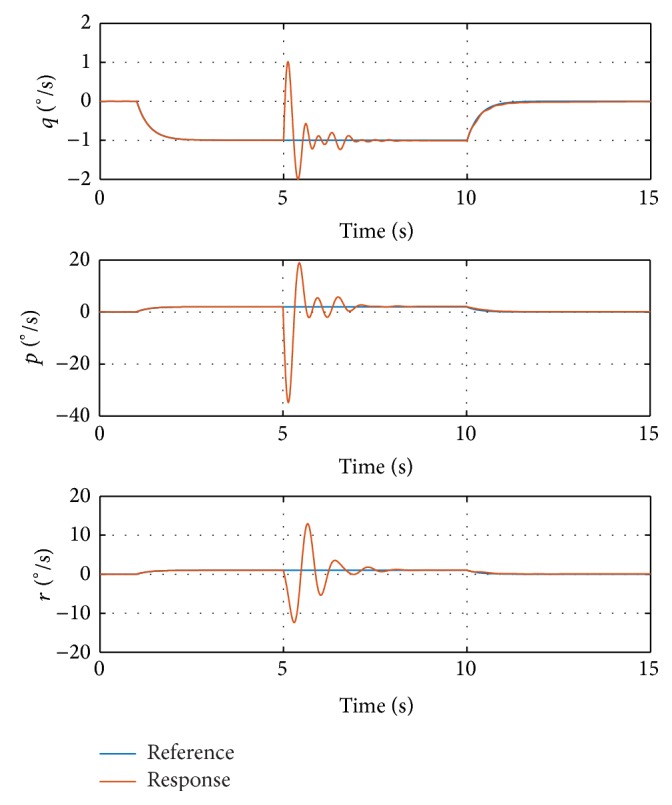
Commands and responses in simulation with step input signal.

The damage case with loss of 20% semispan of the left wing tip is selected. Simulations are conducted on the nonlinear 6DOF GTM model, with maximum deflection of the control surfaces limited within ±30 degrees. The aircraft is flying normally at the beginning, injected with wing tip damage at 5 s during simulation. The simulation results with minor step commands are shown in Figure 3.

**Figure 4:**
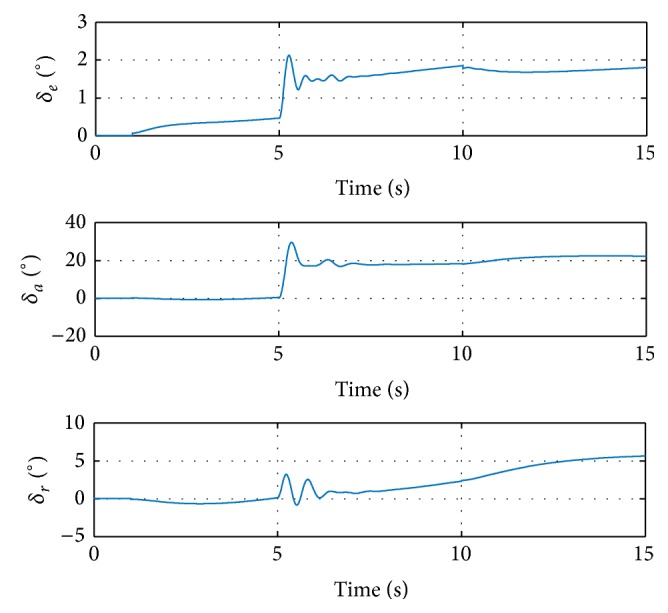
Control surface deflections in simulation with step input signal.

The step input starts at 1 second with value [−1,1, 1]. Figure 3 shows that the response of the undamaged aircraft tracks the reference command from 0 to 5 seconds, which benefits from the proper selection of the initial values of *α* and *f*_0_. The damage is injected at 5 seconds, resulting in some transient oscillations during 5–8 seconds. The magnitudes of the oscillations are relatively small and admissible. After the oscillations, the angular rate responses track the reference signals again. Another step input is injected at 10 seconds. The response signal tracks the reference consistently, which proves the performance of the proposed method. The deflections of control surfaces are shown in Figure 4.

**Figure 5:**
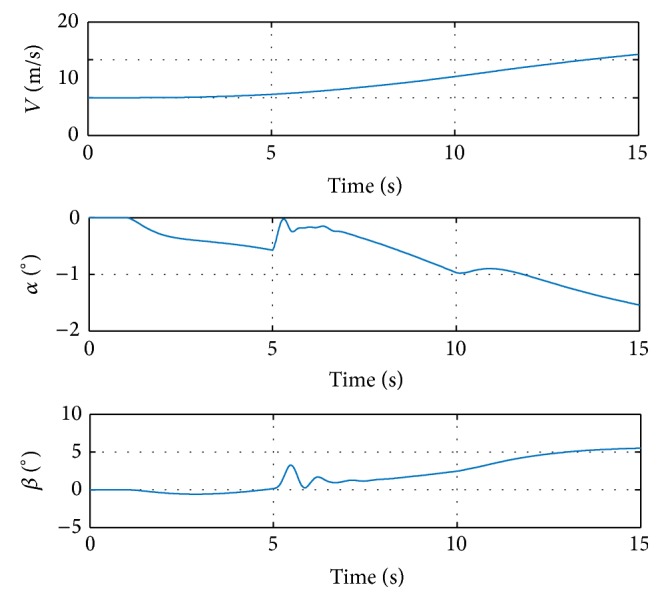
Response of the other states in simulation with step input signal.

Figure 4 shows that all the control surfaces are not saturated; thus, the angular rates are controllable. More explanations can be found in the responses of state *z* in (21), shown in Figure 5.

It can be seen that the responses of the other states are relatively small, especially for the angle of attack *α*. As in Remark 5, (23) indicates that positive *α* with offset *f*_0_ can saturate the ailerons easily, meaning that inappropriate command might cause loss of stability. If the input is selected as [*q*, *p*, *r*] = [1,2, 0], simulation shows that positive *α* will be generated, leaving the ailerons saturated, the response divergent, and the angular rates uncontrollable. Thus, the pitch angular rate command in this case is selected as −1°/s to obtain the negative *α* response, which helps reduce the deflections of control surfaces and results in a controllable aircraft.

**Figure 6:**
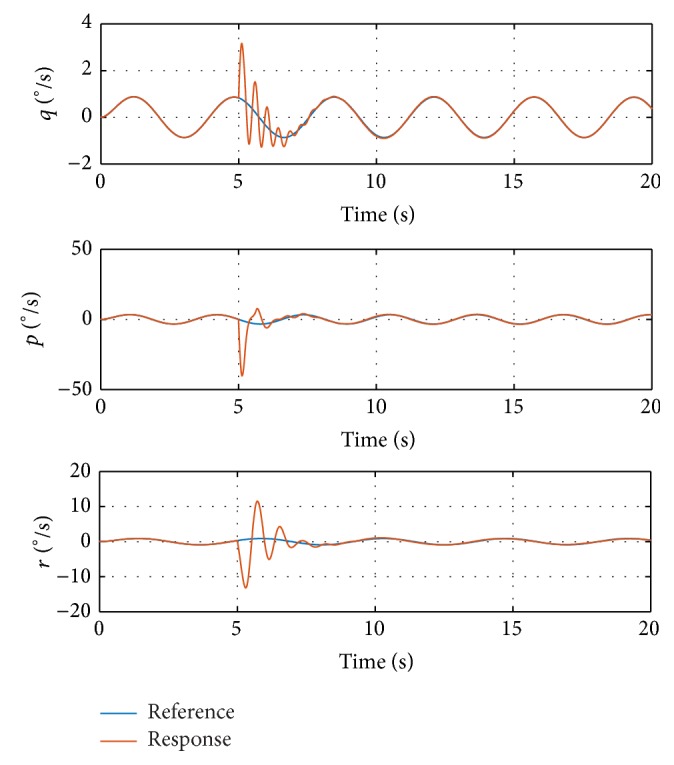
Commands and responses in simulation with sinusoidal input signal.

Further demonstration of tracking performance can be shown in sinusoidal command signals, as in Figure 6.

The sinusoidal inputs are arbitrarily chosen as $q_{c}=sin\sqrt{3}t$, *p*_*c*_ = 4sin2*t*, and $r_{c}=sin\sqrt{2}t$. The damage is injected as 20% left wing tip loss at 5 seconds. As in the step input case, the angular rates track the reference signals before damage, which validates the initial control parameters and the control structure. The transient oscillation lasts from 5 to 8 seconds in the simulation, the magnitude of which is relatively small and admissible. The transient ends at about 8 seconds and the response signals track the reference again. It can be concluded that the proposed MRAC method can restore the handling qualities after the structural damage.

**Figure 7:**
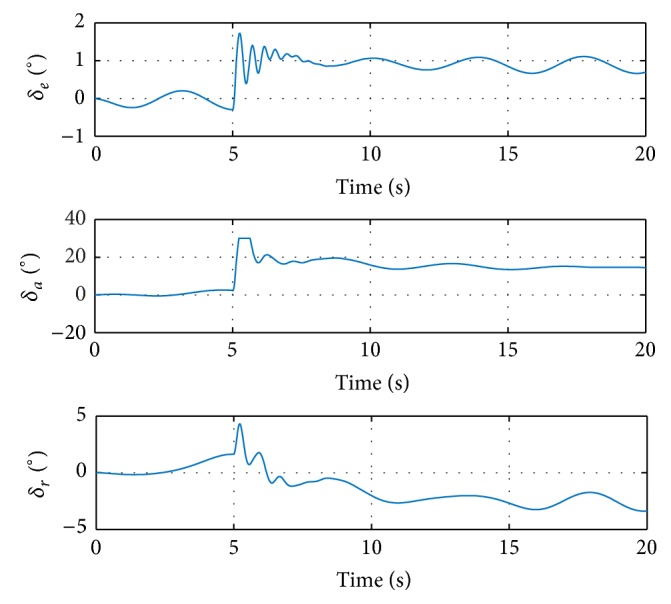
Control surface deflections in simulation with sinusoidal input signal.

The deflections of control surfaces are shown in Figure 7.

**Figure 8:**
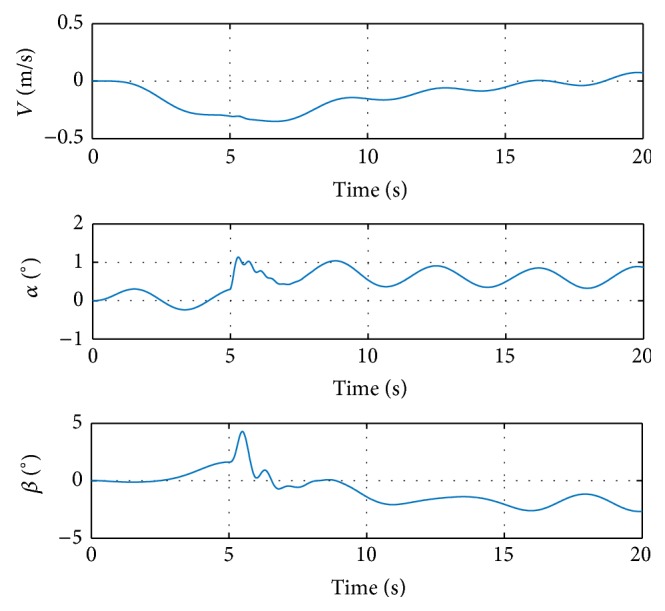
Response of the other states in simulation with sinusoidal input signal.

It can be seen that the ailerons saturate shortly in the oscillations. However, the ailerons come back to about 18 degrees after the transient, and the saturation does not affect the overall performance of the design. The responses of *z* are shown in Figure 8.

It can be seen that the airspeed and air flow angles vary in the same sinusoidal pattern as that in the angular rates. The overall magnitude of state *z* is relatively small, which helps reduce the deflections of control surfaces, making it possible to track the reference signals.

In summary, the simulations show that the proposed MRAC method guarantees error convergence and stability in both step input and sinusoidal input. It can be concluded that the proposed method is validated by simulation.

## 5. Conclusions

In this paper, an MRAC method with prior model knowledge for asymmetric damaged aircraft has been developed. The method is designed for a series of linearized GTM models with the same operation points but with different left outboard wing tip damage degrees. A polytope model for damaged aircraft with small amount of vertices is obtained first. By representing the vast models of various damage cases in a compact tensor-product form, the number of vertices is reduced by omission of smaller singular values using HOSVD algorithm. An extra constant offset term to the derivatives of states is introduced since the actual trimming values of damaged aircraft are unknown. By interpolating the vertex controllers, the model reference control can be achieved. An adaptive law is deduced to update the interpolation coefficients and the trimming values, finalizing the controller design. The method is theoretically proved with closed-loop stability and asymptotic tracking error convergence. Simulations on a nonlinear GTM with damage validate the design.

Some problems remain unsolved in this paper. It was assumed that the *B* matrix is constant before and after damage, but actually it varies a bit. The result is a small tracking error in the simulation of sinusoidal inputs. Future research will address the various assumptions above. The performance of the design is expected to be improved.

## References

[1] Belcastro C. M., Foster J. V. Aircraft loss-of-control accident analysis.

[2] NASA (2006). *NASA Dryden Fact Sheet—Intelligent Flight Control System*.

[3] NASA (2011). *Aviation Safety Program Fact Sheet*.

[4] Bosworth J. T., Williams-Hayes P. S. (2009). Stabilator failure adaptation from flight tests of NF-15B intelligent flight control system. *Journal of Aerospace Computing, Information and Communication*.

[5] Kaneshige J., Burken J. Enhancements to a neural adaptive flight control system for a modified F-15 aircraft.

[6] Yucelen T., Calise A. J. (2011). Derivative-free model reference adaptive control. *Journal of Guidance, Control, and Dynamics*.

[7] Gregory I. M., Cao C., Xargay E., Hovakimyan N., Zou X. L1 adaptive control design for NASA AirSTAR flight test vehicle.

[8] Gregory I., Gadient R., Lavretsky E. Flight test of composite model reference adaptive control (CMRAC) augmentation using NASA AirSTAR infrastructure.

[9] Lavretsky E. Combined/Composite model reference adaptive control.

[10] Crespo L. G., Matsutani M., Annaswamy A. M. Design of a model reference adaptive controller for an unmanned air vehicle.

[11] Gadient R., Levin J., Lavretsky E. Comparison of model reference adaptive controller designs applied to the NASA Generic Transport Model.

[12] Dorobantu A., Murch A., Balas G. H-infinity robust control design for the NASA AirSTAR flight test vehicle.

[13] Cunningham K., Cox D. E., Murri D. G., Riddick S. E. A piloted evaluation of damage accommodating flight control using a remotely piloted vehicle.

[14] Kim B. S., Calise A. J. (1997). Nonlinear flight control using neural networks. *Journal of Guidance, Control, and Dynamics*.

[15] Nguyen N., Krishnakumar K., Kaneshige J., Nespeca P. (2008). Flight dynamics and hybrid adaptive control of damaged aircraft. *Journal of Guidance, Control, and Dynamics*.

[16] Chowdhary G., Johnson E. N., Chandramohan R., Kimbrell M. S., Calise A. (2013). Guidance and control of airplanes under actuator failures and severe structural damage. *Journal of Guidance, Control, and Dynamics*.

[17] Yucelen T., Calise A. J. (2014). Robustness of a derivative-free adaptive control law. *Journal of Guidance, Control, and Dynamics*.

[18] Cao C., Hovakimyan N. (2008). Design and analysis of a novel L1 adaptive control architecture with guaranteed transient performance. *IEEE Transactions on Automatic Control*.

[19] Liu Y., Tao G., Joshi S. M. (2010). Modeling and model reference adaptive control of aircraft with asymmetric damage. *Journal of Guidance, Control, and Dynamics*.

[20] Guo J., Tao G., Liu Y. (2011). Multivariable adaptive control of NASA generic transport aircraft model with damage. *Journal of Guidance, Control, and Dynamics*.

[21] Ouellette J. A. (2010). *Flight dynamics and maneuver loads on a commercial aircraft with discrete source damage [M.S. thesis]*.

[22] Bacon B. J., Gregory I. M. General equations of motion for a damaged asymmetric aircraft.

[23] Qian S., Gang T., Jiaxing G. Multivariable state feedback for output tracking MRAC for piecewise linear systems with relaxed design conditions.

[24] Baranyi P. (2004). TP model transformation as a way to LMI-based controller design. *IEEE Transactions on Industrial Electronics*.

[25] Ioannou P. A., Sun J. (1995). *Robust Adaptive Control*.

[26] Nguyen N., Krishnakumar K., Boskovic J. An optimal control modification to model-reference adaptive control for fast adaptation.

[27] Stepanyan V., Krishnakumar K. (2012). Adaptive control with reference model modification. *Journal of Guidance, Control, and Dynamics*.

[28] Lavretsky E., Gadient R., Gregory I. M. (2010). Predictor-based model reference adaptive control. *Journal of Guidance, Control, and Dynamics*.

[29] Gibson T. E., Annaswamy A., Lavretsky E. (2013). On adaptive control with closed-loop reference models: transients, oscillations, and peaking. *IEEE Access*.

[30] Yucelen T., de la Torre G., Johnson E. N. (2014). Improving transient performance of adaptive control architectures using frequency-limited system error dynamics. *International Journal of Control*.

[31] Yucelen T., Haddad W. M. (2013). Low-frequency learning and fast adaptation in model reference adaptive control. *IEEE Transactions on Automatic Control*.

[32] Asadi D., Sabzehparvar M., Atkins E. M., Talebi H. A. (2014). Damaged airplane trajectory planning based on flight envelope and motion primitives. *Journal of Aircraft*.

[33] Yi G., Zhong J., Atkins E. M., Wang C. (2014). Trim state discovery with physical constraints. *Journal of Aircraft*.

[34] Murch A. A flight control system architecture for the NASA AirSTAR flight test infrastructure.

